# The association of thyroid autoimmunity with ovarian reserve in women with type 1 diabetes with and without polycystic ovary syndrome

**DOI:** 10.1038/s41598-024-63741-1

**Published:** 2024-06-08

**Authors:** Agnieszka Łebkowska, Anna Krentowska, Agnieszka Adamska, Aleksandra Uruska, Anita Rogowicz-Frontczak, Aleksandra Araszkiewicz, Katarzyna Ożegowska, Monika Leśniewska, Paweł Sowa, Ewa Wender-Ożegowska, Dorota Zozulińska-Ziółkiewicz, Irina Kowalska

**Affiliations:** 1 Department of Internal Medicine and Metabolic Diseases, Medical University of Bialystok, M. Sklodowskiej-Curie 24a, 15-276 Bialystok, Poland; 2 Department of Endocrinology, Diabetology and Internal Medicine, Medical University of Bialystok, Bialystok, Poland; 3 Department of Internal Medicine and Diabetology, Poznan University of Medical Sciences, Poznan, Poland; 4 Department of Infertility and Reproductive Medicine, Poznan University of Medical Sciences, Poznan, Poland; 5 Department of Reproduction and Gynaecological Endocrinology, Medical University of Bialystok, Bialystok, Poland; 6 Department of Population Medicine and Lifestyle Diseases Prevention, Medical University of Bialystok, Bialystok, Poland; 7 Department of Reproduction, Poznan University of Medical Sciences, Poznan, Poland

**Keywords:** Endocrinology, Medical research

## Abstract

The aim of the study was to investigate the relation between thyroid autoimmunity (TAI), reflected as the presence of thyroid peroxidase antibodies (TPOAb), and parameters of ovarian reserve in women with type 1 diabetes (T1DM) and polycystic ovary syndrome (PCOS). We studied 83 euthyroid women with T1DM (age – 26 ± 5 years, BMI – 24 ± 3 kg/m^2^) – 12 with PCOS and positive TPOAb (PCOS + TPOAb), 29 with PCOS with negative TPOAb (PCOS + noTPOAb), 18 without PCOS with positive TPOAb (noPCOS + TPOAb), 24 without PCOS with negative TPOAb (noPCOS + noTPOAb). Serum concentrations of anti-Müllerian hormone (AMH), sex hormones, TSH, thyroid hormones and TPOAb were assessed. The prevalence of TAI was comparable between PCOS and noPCOS. We did not observe differences in hormonal profile or AMH concentration between two PCOS groups—PCOS + TPOAb and PCOS + noTPOAb (p > 0.05). Women with PCOS + TPOAb had lower FSH concentration and higher LH/FSH index than noPCOS + noTPOAb (p = 0.027; p = 0.019, respectively). Moreover, PCOS + TPOAb had lower oestradiol level than noPCOS + TPOAb (p = 0.041). AMH concentration was higher in both groups with PCOS, independent of TPOAb presence, than in noPCOS + noTPOAb (both p < 0.001). The presence of positive TPOAb titre was not related to the studied parameters of ovarian reserve – AMH and ovarian follicle number. In multiple linear regression analysis, the only significant predictor of AMH in the whole studied group with T1DM was total daily insulin dose U/kg (β = − 0.264; p = 0.022). The presence of TAI did not affect the hormonal profile or ovarian reserve in women with T1DM with and without PCOS.

## Introduction

The prevalence of polycystic ovary syndrome (PCOS) in women with type 1 diabetes (T1DM) is higher than in general population. A recently published meta-analysis reported the pooled prevalence at the level of 24%, the lowest of 7% observed in Italian adolescents and the highest of 41% in Chilean adolescents and adult women when Rotterdam criteria were used^1^. Hyperglycaemia, autoimmune effects on ovaries, and depletion of ovarian reserve in T1DM makes this group of women at risk of fertility disorders with shorter reproductive time and early menopause^2^. Co-existence with PCOS increases difficulties in diagnostics and treatment encountered in clinical practice. The pathogenesis of PCOS in T1DM women is still not elucidated. The initially suggested link between exogenous insulin and PCOS has not been confirmed yet^1,3^. However, premenarchal onset of T1DM and longer diabetes duration are factors associated with hyperandrogenism and polycystic morphology of ovaries (PCOM)^4^.

Thyroid autoimmunity (TAI) is the most common disorder associated with T1DM, with higher prevalence among women^5^. The best serological markers of TAI, serum thyroid peroxidase antibodies (TPOAb), are present in approximately 95% of patients with TAI^6^. An increasing body of evidence suggests the relationship between TAI and PCOS, showing the prevalence of TAI in PCOS women between 18 and 40%, depending on the diagnostic criteria and ethnicity^7^. A recent analysis of a group of infertile women revealed the presence of TPOAb in 14.5% of them^8^. The possible impact of TPOAb on ovarian function has been confirmed by the presence of these antibodies in ovarian follicular fluid of women with TAI and thyroid peroxidase presence in the mature ovarian follicle^9,10^.

Ovarian reserve is the capacity of ovaries for reproduction. Anti-Müllerian hormone (AMH) is a hormone produced by granulosa cells of early developing follicles and is related to number of primordial follicles, thus is a suitable biomarker for predicting ovarian function in premenopausal women^11^. A recently published meta-analysis underlined that women with T1DM might be in a risk group for decreased AMH level^12^. Supporting this in an earlier large study, lower antral follicle count and AMH level in T1DM women in comparison to healthy controls have been reported^13^. However, in women with PCOS and T1DM, similar to PCOS women without T1DM, AMH concentration is elevated, with accompanying typical ovarian morphology and hyperandrogenism, as a result of its predominant expression by small follicles^14^. In autoimmune disorders, human ovary is usually the target of autoimmune attack, which may lead to progressive ovarian dysfunction. Some studies support the link between TAI and the risk of premature ovarian insufficiency^15^. On the contrary, other authors implicate that TAI and hypothyroidism are not associated with low ovarian reserve, reflected by AMH^16^. In women with T1DM, PCOS and TAI, this subject is still not sufficiently explored.

Considering the above data, our study aimed to elucidate the relation of TAI with markers of ovarian reserve and hormonal profile in women with T1DM, with and without PCOS.

## Materials and methods

### Subjects

The study group consisted of 83 women with T1DM—41 with PCOS and 42 without PCOS. The patients were recruited in two diabetes centres – Department of Internal Medicine and Metabolic Diseases of the Medical University of Bialystok and Department of Internal Medicine and Diabetology of the Poznan University of Medical Sciences, as previously published^4^. The inclusion and exclusion criteria have been described in detail previously^4^.

According to the presence of positive TPOAb antibodies, the patients were divided into four groups: 12 women with PCOS and TPOAb (PCOS + TPOAb), 29 women with PCOS without TPOAb (PCOS + noTPOAb), 18 women without PCOS with TPOAb (noPCOS + TPOAb) and 24 women without PCOS without TPOAb (noPCOS + noTPOAb). The treatment with L-thyroxin was reported by six women in PCOS + TPOAb, five women in noPCOS + TPOAb, one woman in PCOS + noTPOAb, and three women in noPCOS + noTPOAb. Informed written consent has been obtained from each participant after full explanation of the purpose and nature of all procedures.

### Study protocol

A detailed history of diabetes and insulin requirement – mean daily insulin dose and total daily insulin dose per kilogram of body weight per day (TDI)—were obtained in each subject^4^. Physical examination, anthropometric measurements—body mass index (BMI) and waist circumference—were performed as previously described^4^. All signs of clinical hyperandrogenism (presence of hirsutism or acne), menstrual disturbances, and ovarian morphology were evaluated according to Rotterdam ESHRE/ASRM PCOS Consensus Workshop Group diagnostic criteria^17^.

Blood samples were obtained either during the follicular phase (3rd–5th day) or independently of cycle phase in the presence of amenorrhea, collected in both centres and frozen at − 80 °C for further analysis. All blood measurements were performed in the Medical University of Bialystok.

Serum levels of follicle-stimulating hormone (FSH), luteinizing hormone (LH), total testosterone (T), sex hormone–binding globulin (SHBG) and glycated hemoglobin (HbA1c) were measured as previously described^4^. Prolactin and oestradiol concentrations were assessed with the immunoradiometric method (DIAsource ImmunoAssays S.A., Belgium).

Serum TSH concentration (sensitivity 0.025 µIU/ml; intra-assay CV- 0.6%; inter-assay- CV 2.1%), serum fT3 (sensitivity 0.3 pg/ml; intra-assay CV- 6.4%; inter-assay CV- 5.5%) and fT4 (sensitivity 0.03 ng/dl; intra-assay CV—10.3%, inter-assay CV—7.6%) concentrations were detected with radioimmunoassay kits (DIAsource ImmunoAssays S.A., Belgium). Euthyroidism was defined as having normal levels of TSH (reference range, 0.35–4.5 µIU/ml), fT3 (reference range, 2.3–4.2 pg/ml), and fT4 (reference range, 0.89–1.76 ng/dl). TPOAb concentrations were measured with radioimmunoassay kits (ThermoFisher Scientific, Germany) (sensitivity 5.5 U/mL; intra-assay CV—3.9%; inter-assay CV—4.1%). TPOAbs were considered positive if their titre exceeded 60 U/ml. Serum AMH concentrations were determined by enzyme immunoassay (Beckmann Coulter, USA). The lowest concentration of AMH in a sample that could be detected with a 95% probability was 0.08 ng/ml. The intra-assay and inter-assay CVs were below 5.4% and 5.6%, respectively. LH to FSH index (LH/FSH) and free androgen index (FAI) using the formula Tx100/SHBG were calculated.

### Ultrasonography of the thyroid gland

Ultrasound of the thyroid gland was performed with the use of a 7.5 MHz linear transducer (Philips HD5 Diagnostic Ultrasound System, Bothell, Washington, USA, Neusoft Park, Hun Nan Industrial Area, Shenyang 110179, China). Thyroid volume (TV) was calculated using the following equation: (length x width x thickness of the lobes) × 0.479^18^. In each centre, ultrasound was performed by one endocrinologists.

### Ultrasonography of the ovaries

Ultrasound scans of ovaries were done for all the patients by one gynaecologist in each centre with a 5–9 MHz transvaginal transducer (Voluson 730 Expert GE Healthcare) in the early follicular phase of the menstrual cycle. Ovarian volume was calculated using the simplified formula for a prolate ellipsoid^19^. Ovarian volume (O-V) and follicle number (O-FN) in the right and left ovary were calculated and summarized for both ovaries.

### Statistical analysis

Statistical analyses were performed using the Statistica 13.0 package (Statsoft Inc., OK, USA) and Stata/IC 12.1 (StataCorp LP). The variables were tested for normal distribution using the Shapiro–Wilk test. Due to the non-normal distribution of the data, all values were expressed as median (interquartile range). Differences between two groups were assessed using the Mann–Whitney *U* test. Differences between four groups were distinguished by Kruskal–Wallis test with Dunn post hoc test. For categorical variables, the chi-squared test with Fisher exact test was performed. The analysis of correlations between the studied variables was performed using the Spearman test. Additionally, stepwise backward linear regression was applied to evaluate the impact of selected variables on AMH concentration. A p-value < 0.05 was considered statistically significant.

### Ethics approval

All procedures performed in the study were in accordance with the 1964 Helsinki declaration and its later amendments. The study was approved by the Institutional Review Board (Ethics Committee of Medical University of Bialystok, Bialystok, Poland, approval no. R-I-002/300/2015).

## Results

### Study groups

Positive titre of TPOAb was detected in 29% of PCOS women and 42% of women without PCOS, what was not significantly different (p > 0.05).

Table 1 presents the clinical and biochemical characteristics of the studied groups. Women in the four groups did not differ significantly in age and anthropometric measurements (p > 0.05). The median of T1DM duration was 10 years in the whole studied group and was not different between the subgroups (Table 1). HbA1c was lower in PCOS + noTPOAb than noPCOS + noTPOAb (p = 0.021).

**Table 1:** Clinical and hormonal characteristics of T1DM women with and without PCOS, with positive and negative TPOAb.

	PCOS + TPOAb, N = 12	PCOS + noTPOAb, N = 29	noPCOS + TPOAb, N = 18	noPCOS + noTPOAb, N = 24	P-value
Age, years	24 (22–25)	25 (22–29)	27 (25–33)	27 (21–32)	0.213
BMI, kg/m^2^	25 (21.7–26.1)	25 (22.1–26.3)	24.3 (22.8–27.1)	22.9 (21.125.1)	0.416
T1DM duration, years	12 (5.5–13.5)	10 (8–14)	14 (7–19)	9 (5–13)	0.399
Age of menarche, years	12 (11–13)^2^	13 (12–14)	14 (13–15)	13 (12–14)	0.009
Total daily insulin dose, U/kg	0.42 (0.4–0.6)	0.5 (0.5–0.6)	0.6 (0.6–0.7)	0.6 (0.5–0.6)	0.059
HbA1c, %	7.1(6.7–7.9)	7.1 (6.6–8.2)^4^	7.7 (7–8.5)	8.9 (7.3–9.1)	0.014
HbA1c, mmol/mol	54 (50–63)	50 (49–66)	61 (53–69)	74 (56–76)	
LH, mIU/ml	4.7 (3.9–6.2)	4.5 (3.7–5.6)	4 (2.9–5.4)	4.6 (3.1–5.2)	0.551
FSH, mIU/ml	3.8 (3–4.6)^1^	5 (3.6–6.4)	4.9 (4.4–6)	6.2 (4.8–7.8)	0.03
LH/FSH	1.3 (0.8–2.6)^1^	0.9 (0.8–1.4)	0.9 (0.6–1)	0.7 (0.6–0.9)	0.014
Oestradiol, pg/ml	33 (28.8–65.6)^2^	54 (39.5–85.9)	68.3 (51–91.9)	66.4 (32.6–89.5)	0.06
Testosterone, ng/ml	0.7 (0.6–0.8)	0.7 (0.5–1)	0.5 (0.4–0.7)	0.5 (0.4–0.7)	0.026
FAI	3.8 (2.5–6.4)	3.5 (2.4–5.6)	2.4 (1.6–3.5)	2.5 (1.7–3.3)	0.047
SHBG, nmol/l	67 (54.6–74.9)	68 (47.5–83.5)	61.2 (47.2–91.7)	82.4 (48–98.8)	0.623
Prolactin, ng/ml	9.5 (6.2–12.7)	11.2 (8.3–15.1)	8.2 (6.3–9.4)	9.2 (7.3–13.3)	0.244
AMH, ng/ml	7.1 (4.4–9.3)^1^	6.4 (4.1–8.9)^4^	3.9 (2.9–6.8)	2.8 (1.6–4.1)	< 0.001
O-V, cm^3^	19 (16.6–27.9)^1,2^	14.3 (10.7–20.3)	10.2 (9.5–14.1)	10.6 (8.4–15.7)	0.002
O-FN, n	24 (19–29)^1^	21 (18–28)	15 (13–19)	12 (9–17)	< 0.001
TSH, IU/ml	2.7 (1.1–3.6)	1.6 (1.3–2.4)^3,4^	1.3 (1–2.3)	1.6 (0.9–2.5)	0.755
fT4, ng/dl	1.3 (1.2–1.4)	1.2 (1.1–1.3)	1.2 (1.1–1.3)	1.3 (1.2–1.3)	0.494
fT3, pg/ml	3.2 (3–4)	3 (2.7–3.3)	3 (2.7–3.2)	3.2 (2.6–3.4)	0.295
TPOAb, IU/ml	723 (229–1798)^1,5^	15 (7–26)^3^	828 (197–1510)^6^	24 (7–35)	< 0.001
TV, ml	8.6 (7.2–11)	8.9 (6.9–11.7)	13 (5.9–14.7)	8.2 (6.4–10.7)	0.634

Data are expressed as median (interquartile range).

*BMI* body mass index, *HbA1c* haemoglobin A1c, *FSH* follicle-stimulating hormone, *LH* luteinizing hormone, *FAI* free androgen index, *SHBG* sex hormone binding globulin, *TSH* thyroid‑stimulating hormone, *fT4* free T4, *fT3* free T3, *AMH* anti-Müllerian hormone, *O-V* ovarian volume, *O-FN* ovarian follicle number, *TV* thyroid volume.

^1^p < 0.05 PCOS + TPOAb vs noPCOS + noTPOAb.

^2^p < 0.05 PCOS + TPOAb vs noPCOS + TPOAb.

^3^p < 0.05 PCOS + noTPOAb vs noPCOS + TPOAb.

^4^p < 0.05 PCOS + noTPOAb vs noPCOS + noTPOAb.

^5^p < 0.05 PCOS + TPOAb vs PCOS + noTPOAb.

^6^p < 0.05 noPCOS + TPOAb vs noPCOS + noTPOAb.

All subjects were in euthyroidism. TSH levels and fT4 and fT3 levels were comparable between the studied groups, independent of TPOAb presence. There were also no differences in TV among the groups.

### Clinical and hormonal characteristics

PCOS + TPOAb and PCOS + noTPOAb had comparable concentrations of LH, FSH, T, and PRL. PCOS + TPOAb experienced earlier menarche than noPCOS + TPOAb (p = 0.006). Women with PCOS + TPOAb had lower FSH concentration and higher LH/FSH index than noPCOS + noTPOAb (p = 0.027; p = 0.019, respectively). Moreover, PCOS + TPOAb had lower oestradiol level than noPCOS + TPOAb (p = 0.041). The median of AMH concentration was higher in PCOS + TPOAb and PCOS + noTPOAb in comparison to noPCOS + noTPOAb (both p < 0.001). O-V was significantly higher in PCOS + TPOAb than in both noPCOS groups with and without TPOAb (p = 0.005 and p = 0.006, respectively). O-FN was higher in both PCOS + TPOAb and PCOS + noTPOAb when compared to noPCOS + noTPOAb (p = 0.004, p < 0.001), and in PCOS + noTPOAb compared to noPCOS + TPOAb (p = 0.035).

### Relationship between ovarian reserve and TAI

Spearman correlations among all studied women showed that the TPOAb titre did not correlate with the parameters of ovarian reserve – AMH and O-FN. LH/FSH index correlated positively with AMH level and O-FN (r = 0.278, p = 0.015; r = 0.324, p = 0.005 respectively).

**Figure 1:**
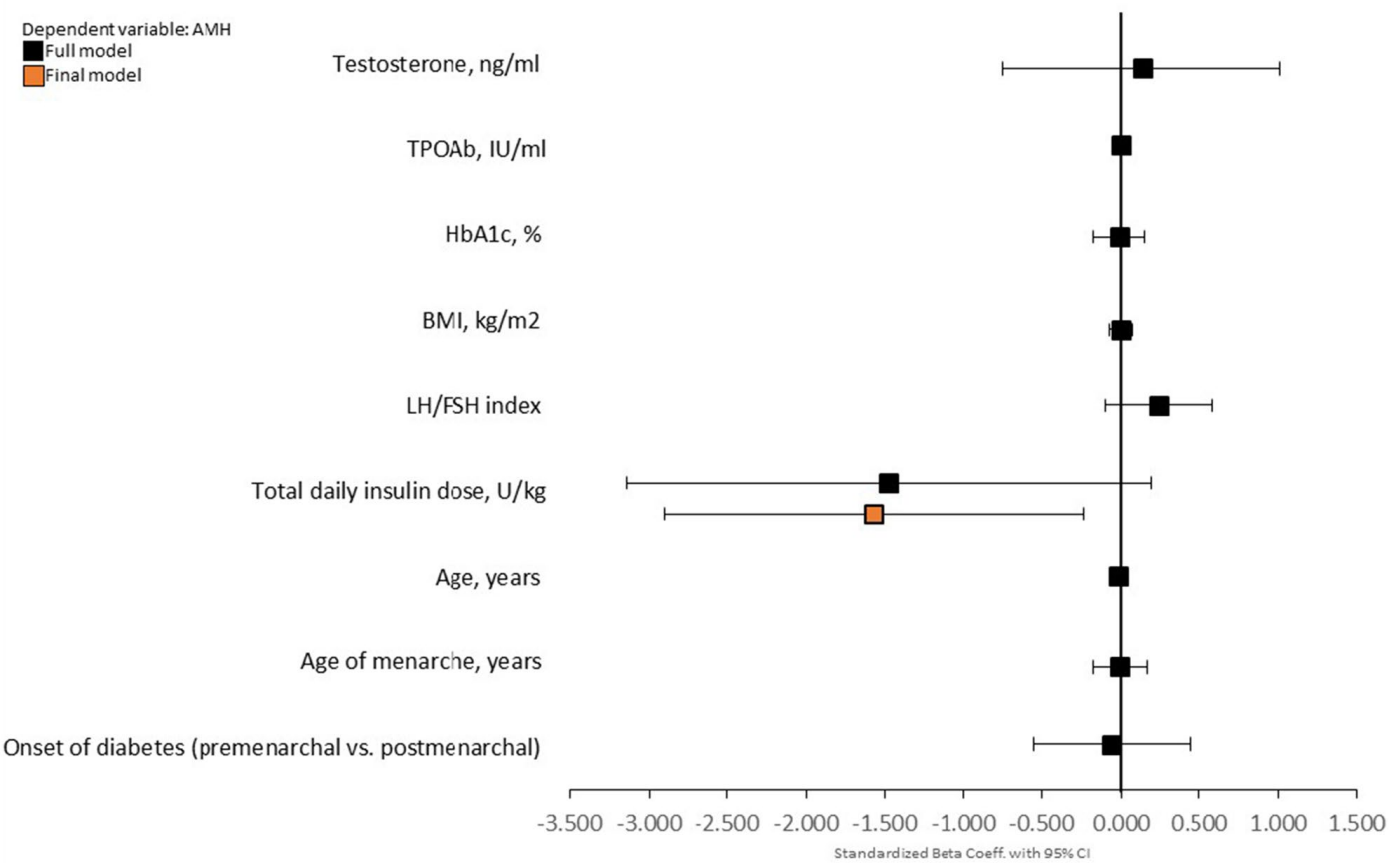
The association of selected variables with serum AMH concentration in the whole studied group in backward stepwise linear regression analysis. *BMI* body mass index, *TPOAb* thyroid peroxidase antibodies, *LH/FSH* luteinizing hormone to follicle-stimulating hormone index, *HbA1c* haemoglobin A1c.

To assess the influence of the studied variables on AMH concentration, we performed multiple linear regression analysis. It has been found that the TPOAb titre had no influence on AMH level. The only significant predictor of AMH in the whole studied group with T1DM was TDI (β = − 0.264; p = 0.022). The full results of the analysis are shown in Fig. 1.

## Discussion

In this study we analysed women with T1DM with and without PCOS, focusing on the possible impact of TAI on parameters of ovarian reserve—hormonal status and ovarian parameters. The comparison of four groups revealed differences resulting from PCOS characteristics with increased level of AMH in both PCOS groups than in noPCOS + noTPOAb. Positive titre of TPOAb did not influence AMH concentration and ovarian parameters among the whole studied group. Interestingly, the only significant predictor of AMH level was the insulin dose.

The incidence of PCOS in T1DM women is higher than in general population. In a recently published study in young women from India with median T1DM duration of 12 years, 52% of them had PCOM, 26% hyperandrogenism, and 27% fulfilled PCOS criteria^20^. An excellent diagnostic marker of PCOM is AMH. AMH is produced by growing follicles from the primary up to the small antral follicles and has a double negative function in ovary. It inhibits the recruitment of primordial follicles and reduces the sensitivity of antral follicles to FSH, separating the FSH-independent and FSH-dependent phases of folliculogenesis^21^. Generally, there is evidence for decreased AMH levels in women with T1DM when compared to healthy controls, presented in a recently published meta-analysis^12^. On the other side, discussion on PCOS and increased AMH levels among women with PCOS and T1DM continues^14^. Despite the suggested potential hypothesis regarding the influence of exogenous insulin, acting as a co-gonadotrophin in ovaries, the pathogenesis is still not fully elucidated. Our previous study confirmed the relation of premenarchal T1DM diagnosis, time of the beginning of insulin therapy, and T1DM duration with hyperandrogenism and PCOM^4^. What is important, AMH concentration of 3.74 ng/ml can be the predictor of PCOS in T1DM patients with 90.2% sensitivity and 70.3% specificity^14^.

The published data suggested a possible link between thyroid function and ovaries. Firstly, the presence of thyroid hormone receptors in granulosa cells has been confirmed^22^. The increasing expression of these receptors with stages of follicle maturation from primordial and primary follicles to the mature oocyte suggested that triiodothyronine influences the oocyte maturation. Additionally, the presence of type 2 and 3 deiodinase in granulosa cells enable thyroxine conversion to triiodothyronine^22^. Finally, the presence of thyroid peroxidase in human cumulus granulosa cells has been detected lately^9^. These findings indicate that human ovarian follicle can be dependent on the metabolism of thyroid hormones. However, the presence of TPOAb in TAI may disrupt the function of thyroid peroxidase and then maturation of the human oocyte.

In the current study, we focused on TAI impact and its relations with ovarian reserve. The association between TAI and ovarian reserve has been described in several studies, although the data are inconsistent, and the studies were performed in populations without T1DM. In the literature, the presence of TAI in women with euthyroidism or subclinical hypothyroidism, regardless of levothyroxine treatment, was a significant predictor of pregnancy loss, comparing to women without thyroid autoantibodies^23^. In a large cross-sectional study by Polyzos et al., TAI and hypothyroidism were not associated with low ovarian reserve expressed by low age-adjusted AMH values among women from the general population^16^. However, lower FT3 and positive TPOAb titre were associated with lower antral follicle count in women with diminished ovarian reserve and infertility^24^. Some authors suggest that PCOS might be considered an autoimmune disease with positive titres of organ- and nonorgan-specific autoantibodies^25^. Clinical reports have shown that women with TAI and PCOS had lower fertility rate, lower AMH concentration, and higher risk of clomiphene citrate resistance than women with PCOS but without TAI^26,27^. Moreover, AMH level was negatively related to titre of TPOAb in PCOS group, independent of PCOS phenotype^28^. In contrast, one study assessing serum concentration of AMH in TPOAb-positive and negative PCOS patients did not reveal differences between the groups^29^. In our study in T1DM women, we did not notice different prevalence of TAI between PCOS and noPCOS. It is probably due to the strong co-existence of T1DM with other autoimmune diseases, mainly TAI and celiac disease^30^.

There is still a debate regarding possible factors affecting ovarian reserve in women with T1DM with and without PCOS. Our results did not confirm the influence of TPOAb presence on AMH value in women with T1DM. Similar prevalence of TAI in both PCOS and noPCOS groups allow us to suggest that the presence of TAI is not a significant factor determining ovarian reserve in this group of women. Other published reports in groups of T1DM patients gave us the suggestion that the methods of insulin administration or dose of long-acting insulin might have an impact on PCOS and AMH concentration^14,31^. A meta-analysis of PCOS in T1DM underlined that systemic hyperinsulinism may play a role in the development of ovarian disturbances, although individual predispositions and other unknown factors should be taken into consideration^1^. However, the observation of T1DM women with median age of 35 years in Epidemiology of Diabetes Interventions and Complications study (EDIC) revealed that elevated AMH level and PCOM presence were observed in one-third of the studied population. Factors such as lower insulin dose, younger age, not smoking, and high T concentrations were associated with AMH level^32^. This is consistent with our study, in which we found that TDI was negatively related to AMH concentration in the whole studied group of women younger than in EDIC, with median age of 26, but with comparable duration of T1DM. A prospective 17-year observation of selected group of women with T1DM in EDIC gave us evidence that AMH concentrations decline in a manner similar to women without T1DM and that it was not associated with time-weighted insulin dose^13^. There are suggestions that insulin may not act as co-gonadotrophins in late reproductive age. However, larger studies are needed to clarify these mechanisms.

Analysing other variables connected with ovarian function, we observed decreased FSH concentration in PCOS comparing to noPCOS, independent of TPOAb presence, and lower oestradiol concentration in PCOS + TPOAb group, comparing to noPCOS + TPOAb. Additionally, we found that LH/FSH index was correlated with AMH and O-FN. In a meta-analysis of studies in women with T1DM focusing on ovarian reserve, diminished FSH concentration also has been noticed but without correlations with AMH. However, the authors showed that oestradiol level was negatively correlated with daily insulin doses and HbA1c^12^. It is known that gonadotrophins have impact on AMH production. High level of AMH in PCOS is connected with overproduction of androgens under the impact of LH^33^. Similar possible explanation would be in T1DM, when insulin enhances LH effects on theca cells, leading to increased recruitment and growth of antral follicles^21^. The inhibited FSH level in the follicular phase of cycle, observed in our study, supports the role of AMH in the regulation of follicle growth initiation and setting the threshold for FSH sensitivity before ovulation^33^. What is more, there is evidence in PCOS that AMH decreases aromatase activity in granulosa cells, reducing oestradiol production^33^. On the other side, there is also connection of high oestrogen level with autoimmunity in PCOS, as Arduc et al. found that serum levels of oestradiol were higher in TPOAb-positive PCOS women than TPOAb-negative ones^34^. However, our study did not reproduce this finding.

There are several limitations of the present study. The first one is a relatively small number of study participants. Also, the assessment of only TPOAb, mostly associated with TAI, do not address wider autoimmunity. We did not specify the AMH levels according to age. There is no information on duration of TAI, age of TAI occurrence and the time in relation to menarche, which could help interpret the data. There was also lack of comparison of the studied groups with women without T1DM. However, our study was performed in women within euthyroid range to exclude the effects of hypothyroidism and in the follicular phase as necessary in hormonal data analysis.

## Conclusions

The prevalence of TAI among women with T1DM is comparable between groups with and without PCOS. Positive titre of TPOAb was not related to AMH level, ovarian morphology, or phenotype of PCOS in women with T1DM. The only predictor of AMH concentration in women with T1DM was lower insulin dose. Rising prevalence of PCOS in T1DM makes the management of women in reproductive age challenging. Thus, predictors of ovarian reserve and its decline still require further studies.

## Data Availability

The data supporting the findings of this study are available from the corresponding author on reasonable request.
